# Hyponatremia in Children and Adults with Prader–Willi Syndrome: A Survey Involving Seven Countries

**DOI:** 10.3390/jcm10163555

**Published:** 2021-08-12

**Authors:** Muriel Coupaye, Karlijn Pellikaan, Anthony P. Goldstone, Antonino Crinò, Graziano Grugni, Tania P. Markovic, Charlotte Høybye, Assumpta Caixàs, Helena Mosbah, Laura C. G. De Graaff, Maithé Tauber, Christine Poitou

**Affiliations:** 1 Assistance Publique-Hôpitaux de Paris, Rare Diseases Center of Reference ‘Prader-Willi Syndrome and Obesity with Eating Disorders’ (PRADORT), Nutrition Department, Pitié-Salpêtrière Hospital, F-75013 Paris, France; helena.mosbah@aphp.fr (H.M.); christine.poitou-bernert@aphp.fr (C.P.); 2 International Network for Research, Management & Education on Adults with Prader-Willi Syndrome; tony.goldstone@imperial.ac.uk (A.P.G.); a.crino@tiscali.it (A.C.); g.grugni@auxologico.it (G.G.); tania.markovic@sydney.edu.au (T.P.M.); charlotte.hoybye@sll.se (C.H.); acaixas@gmail.com (A.C.); l.degraaff@erasmusmc.nl (L.C.G.D.G.); 3 European Reference Network on Rare Endocrine Conditions; l.degraaff@erasmusmc.nl (L.C.G.D.G.); tauber.mt@chu-toulouse.fr (M.T.); 4 Department of Internal Medicine, Division of Endocrinology, Erasmus Medical Center, University Medical Centre Rotterdam, 3015 GD Rotterdam, The Netherlands; k.pellikaan@erasmusmc.nl (K.P.); l.degraaff@erasmusmc.nl (L.C.G.D.G.); 5 Department of Internal Medicine, Division of Endocrinology, Center for Adults with Rare Genetic Syndromes, Erasmus Medical Center, University Medical Center Rotterdam, 3015 GD Rotterdam, The Netherlands; k.pellikaan@erasmusmc.nl (K.P.); l.degraaff@erasmusmc.nl (L.C.G.D.G.); 6 Dutch Center of Reference for Prader-Willi Syndrome, 3015 GD Rotterdam, The Netherlands; k.pellikaan@erasmusmc.nl (K.P.); l.degraaff@erasmusmc.nl (L.C.G.D.G.); 7 Academic Centre for Growth Disorders, Erasmus Medical Center, University Medical Centre Rotterdam, 3015 GD Rotterdam, The Netherlands; k.pellikaan@erasmusmc.nl (K.P.); l.degraaff@erasmusmc.nl (L.C.G.D.G.); 8 PsychoNeuroEndocrinology Research Group, Centre for Neuropsychopharmacology, Division of Psychiatry, and Computational, Cognitive and Clinical Neuroimaging Laboratory, Department of Brain Sciences, Faculty of Medicine, Imperial College London; Department of Endocrinology, Imperial College Healthcare NHS Trust; Hammersmith Hospital, London W12 0NN, UK; 9 Reference Center for Prader-Willi Syndrome, Bambino Gesù Hospital, Research Institute, 00050 Palidoro, Italy; 10 Divison of Auxology, Istituto Auxologico Italiano, IRCCS, 28824 Piancavallo, Italy; 11 Metabolism & Obesity Services, Royal Prince Alfred Hospital, Camperdown, NSW 2050, Australia; 12 Charles Perkins Centre, Faculty of Medicine and Health, University of Sydney, Sydney, NSW 2006, Australia; 13 Department of Molecular Medicine and Surgery, and Department of Endocrinology, Karolinska Institute and Karolinska University Hospital, 171 76 Stockholm, Sweden; 14 Department of Endocrinology and Nutrition, Hospital Universitari Parc Taulí, Institut d’Investigació i Innovació Parc Taulí (I3PT), Universitat Autònoma de Barcelona, 08208 Sabadell, Spain; 15 Rare Diseases Center of Reference ‘Prader-Willi Syndrome and Obesity with Eating Disorders’ (PRADORT), Department of Endocrinology, Bone Diseases, Genetics, and Gynecology, Children’s Hospital, Institut Toulousain des Maladies Infectieuses et Inflammatoires (Infinity) INSERM UMR1291—CNRS UMR5051—Université Toulouse III, F-31000 Toulouse, France; 16 INSERM, Nutrition and Obesities: Systemic Approaches (NutriOmics), Research Unit, Sorbonne University, F-75013 Paris, France

**Keywords:** Prader–Willi syndrome, hyponatremia, excessive fluid intake, syndrome of inappropriate antidiuretic hormone, desmopressin

## Abstract

In Prader–Willi syndrome (PWS), conditions that are associated with hyponatremia are common, such as excessive fluid intake (EFI), desmopressin use and syndrome of inappropriate antidiuretic hormone (SIADH) caused by psychotropic medication. However, the prevalence of hyponatremia in PWS has rarely been reported. Our aim was to describe the prevalence and severity of hyponatremia in PWS. In October 2020, we performed a retrospective study based on the medical records of a large cohort of children and adults with PWS from seven countries. Among 1326 patients (68% adults), 34 (2.6%) had at least one episode of mild or moderate hyponatremia (125 ≤ Na < 135 mmol/L). The causes of non-severe hyponatremia were often multi-factorial, including psychotropic medication in 32%, EFI in 24% and hyperglycemia in 12%. No obvious cause was found in 29%. Seven (0.5%) adults experienced severe hyponatremia (Na < 125 mmol/L). Among these, five recovered completely, but two died. The causes of severe hyponatremia were desmopressin treatment for nocturnal enuresis (*n* = 2), EFI (*n* = 2), adrenal insufficiency (*n* = 1), diuretic treatment (*n* = 1) and unknown (*n* = 1). In conclusion, severe hyponatremia was very rare but potentially fatal in PWS. Desmopressin treatment for nocturnal enuresis should be avoided. Enquiring about EFI and monitoring serum sodium should be included in the routine follow-ups of patients with PWS.

## 1. Introduction

Hyponatremia, defined as a serum sodium concentration below 135 mmol/L, is the most common electrolyte disorder encountered in clinical practice and has affected up to 30% of hospitalized patients across numerous studies throughout the world over the last several decades [1,2]. Severe hyponatremia (<125 mmol/L) can lead to life-threatening neurological symptoms (e.g., confusion, seizures, coma), especially if it occurs rapidly [3]. Several factors that might contribute to hospital-acquired hyponatremia have been identified, including increasing age [1], diuretics, surgery, hypotonic intravenous fluids and several drugs and diseases that promote the release of antidiuretic hormone [4]. Indeed, the syndrome of inappropriate antidiuretic hormone (SIADH) is the most common cause of hyponatremia and occurs when there is persistent secretion of antidiuretic hormone (ADH, also called vasopressin) despite hyponatremia [5,6].

Prader–Willi syndrome (PWS) is a rare genetic neuro-endocrine developmental disorder and the most common form of syndromic obesity with an incidence of approximately 1 in 21,000 newborns [7]. This genetic syndrome is caused by a loss of the expression of paternally inherited imprinted alleles on chromosome 15q11-q13 that can occur via three mechanisms. The main genetic mechanism is a paternal deletion in about 60%, followed by maternal uniparental disomy (mUPD) in 36% and imprinting defects in 4% of the overall PWS population [8]. Impaired hypothalamic development and function are the causes of many of the phenotypes comprising the developmental trajectory of PWS: from anorexia at birth to excessive weight gain preceding hyperphagia and early-onset severe obesity with combined hormonal deficiencies, behavioral problems and dysautonomia [9]. Among the hormonal deficits, hypogonadism is the most frequent in PWS, with expression in both sexes and at all ages [9] and sex hormone substitution could present a greater risk for hyponatremia in women [10]. In addition, central adrenal insufficiency is rare, about 1% in adults with PWS [11], but is known to induce hyponatremia.

Severe hyponatremia in patients with PWS was only reported in three studies involving adults [12,13] and an infant [14], despite universal hypothalamic–pituitary axis dysfunction in PWS, as well as an increased risk of water intoxication by excessive fluid intake (EFI), at least in patients with mUPD [12]. In addition, patients with PWS are frequently exposed to psychotropic medications that are known to cause SIADH (i.e., carbamazepine, tricyclic and selective serotonin reuptake inhibitor (SSRI) antidepressants, phenothiazines, haloperidol) [6]. Moreover, some patients may receive desmopressin treatment (synthetic analog of ADH) to manage nocturnal enuresis, which is observed in PWS [15]. Finally, the possibility of SIADH due to dysfunction of the hypothalamic nuclei engaged in ADH production in patients with PWS was suggested in one study [12] but has not been confirmed clinically.

Recently, four French adults with PWS experienced severe hyponatremia, leading to death in one case, prompting a national survey. However, no other case of severe hyponatremia was reported in adults or children. This led to performing a retrospective cohort study in nine reference centers for PWS of the International Network for Research, Management and Education on adults with PWS (INfoRMEd-PWS) to collect all the cases of hyponatremia. The aim of this study was to describe cases of hyponatremia in patients with PWS that were followed in seven countries and provide clinical recommendations to prevent severe hyponatremia.

## 2. Methods

### 2.1. Study Rationale and Design

The first part was a case series of four severe hyponatremia patients who were investigated using a descriptive clinical study. Then, we conducted a retrospective cohort study based on medical records of nine reference centers for PWS in seven countries constituting the INfoRMEd-PWS. All patients with genetically confirmed PWS had at least one systematic health screening every year, consisting of a medical interview, a complete physical examination and routine biochemical measurements. In October 2020, each center’s investigator reviewed medical data from the last decade of their reference center for PWS to report all cases of patients with hyponatremia (Na < 135 mmol/L). Then, the investigator completed the characteristics of patients with hyponatremia (demography, comorbidities), the possible causes and the management of hyponatremia.

All participating centers (nine reference centers for PWS in seven countries) obtained approval from ethics committees and patients or caregivers were informed about the retrospective analysis of the data.

### 2.2. Statistical Analysis

Data are expressed as mean ± SD (range) or numbers (%). We used descriptive statistics to report the demographic information and medical data of the patients with hyponatremia.

## 3. Results

The survey was based on 1326 patients (430 children and 896 adults) with PWS that were currently or had been (patients lost to follow-up or deceased) under the care of nine reference centers for PWS in seven countries in Europe and Australia: France (265 children and 315 adults), Italy (135 children and 252 adults), the Netherlands (122 adults), Spain (30 children and 50 adults), United Kingdom (46 adults), Sweden (41 adults) and Australia (70 adults).

Among these 1326 patients with PWS, 34 (2.6%) had at least one episode of mild or moderate hyponatremia (125 ≤ Na < 135 mmol/L), including two children (0.02%), and 7 (0.5%) had a severe episode of hyponatremia (Na < 125 mmol/L), all being adults.

### 3.1. Individual Clinical Presentation of the Four Most Recent Cases with Severe Hyponatremia

We first detailed the four most recent and severe French cases from the reference center of Pitié Salpêtrière hospital in Paris.

The first case was a 41-year-old male with mUPD. His routine treatment comprised 12.5 mg of the antipsychotic medication loxapine at bedtime, glucose-lowering medications (gliclazide, metformin, sitagliptin), antihypertensive medication (irbesartan) and allopurinol. He had nocturnal enuresis for several years, for which a urologist initially prescribed 120 µg/day of sublingual desmopressin (Minirin Melt^®^), but since this was ineffective, the dose was doubled to 240 µg/day. Three days later, the patient developed generalized tonic-clonic seizures and was admitted to the intensive care unit (ICU). His serum sodium on admission was 115 mmol/L. His brain computed tomography (CT) scan was normal. Neither urine electrolyte, urine osmolality analysis, measurement of serum cortisol, nor thyroid function tests were performed, as the etiology of the hyponatremia was considered to be undoubtedly related to the desmopressin. The serum sodium returned to normal on day 3 after fluid restriction and administration of oral NaCl. Desmopressin was stopped and there has been no recurrence of hyponatremia.

The second case was a 27-year-old male with mUPD with a history of EFI for 5 years after beginning psychotropic drug therapy (12.5 mg per day of loxapine and 200 mg per day of topiramate). In December 2019, he had an episode of diarrhea for 3 days and suddenly developed generalized tonic-clonic seizures. On admission to the ICU, his serum sodium was 119 mmol/L, urinary osmolality was 169 mOsmol/kg and urinary sodium was 25 mmol/L. There was no thyroid nor cortisol insufficiency. He recovered in 5 days with fluid restriction and the hyponatremia has not recurred to date with fluid restriction (1.5 L/d).

The third case was a 23-year-old female with mUPD. Her only known medication was a growth hormone treatment. She had a history of nocturnal enuresis and was treated with oral desmopressin in childhood (Minirin^®^ tablets). The dose was decreased from 200 to 100 µg daily when she was transferred to the adult nutrition department because her serum sodium concentration was 131 mmol/L. Desmopressin was stopped in January 2020 because of persistent mild hyponatremia and a lack of efficacy. In June 2020, she started to have vertigo, then became comatose and was admitted to ICU. Her serum sodium was 115 mmol/L and her potassium was also low at 3.2 mmol/L (3.5–5.1). There was no thyroid nor cortisol insufficiency. Her mother reported 3 days of EFI before the coma, but the urinary osmolality was high at 482 mOsmol/kg. Urinary sodium was also elevated at 75 mmol/L, consistent with SIADH. After the serum sodium returned to normal with fluid restriction, she admitted that she secretly took desmopressin tablets. She no longer has access to any desmopressin and there has been no recurrence of hyponatremia to date.

The fourth case was a 31-year-old man with mUPD who was admitted to the psychiatry unit in July 2020 because of psychiatric decompensation, which was related to severe anxiety, in part due to the COVID-19 pandemic. After increasing his psychiatric medication (diazepam, clopixol, loxapine, valproic acid), he displayed confusion, for which he required short-term hospitalization. His serum sodium was 122 mmol/L at presentation and he recovered within 48 h with fluid restriction, resulting in the normalization of his serum sodium (139 mmol/L). In November 2020, he slipped and fractured his ankle, for which he had surgery on 11 November 2020. When the cast was removed on 1 December 2020, the scar was noted to be infected and admission for debridement was recommended, but this was delayed and finally scheduled for 21 December 2020. Serum sodium was 135 mmol/L on 18 December 2020. On the morning of 21 December 2020, he was found unconscious in the bathroom and was transferred to ICU. His serum sodium was 112 mmol/L, potassium 4.0 mmol/L (3.5–5.1) and urea 2.3 mmol/L (2.5–7.4). The brain CT scan found a global erasure of cortical furrows without bleeding or signs of ischemia. Despite the correction of hyponatremia, he developed bilateral areactive mydriasis. A second brain CT scan found an increase in diffuse edema with effacement of the basal cisterns and an absence of vascularization in the arterial and venous phase. He died on 24 December 2020. After questioning his mother, it is very likely that he drank copious amounts of water from the bathroom tap in response to anxiety about the forthcoming surgery, which was postponed twice.

### 3.2. Severe Cases of Hyponatremia in PWS

Table 1 shows the seven cases of severe hyponatremia (Na < 125 mmol/L) in adults with PWS from three countries (France, the Netherlands and the United Kingdom), including the four French cases described above. The mean age was 37.2 ± 11.7 years (23–55), the sex ratio was close to 1 (4 males and 3 females) and mUPD was the predominant genetic diagnosis (71%). In two patients, the hyponatremia was due to the desmopressin that was used to control nocturnal enuresis. In four patients, there were several possible contributory factors, and in the remaining patient, there was no apparent cause. All patients with severe hyponatremia were treated in emergency care and then admitted to ICU. Five patients recovered completely but two patients died (one from cerebral edema and one from probable inhalation of gastric content).

### 3.3. Mild or Moderate Cases of Hyponatremia in PWS

Thirty-four cases of moderate or mild hyponatremia were reported from seven countries (Table 2). All patients were asymptomatic (incidental finding), except one who presented with confusion (serum sodium 127 mmol/L), the mean age was 36 years, the sex ratio was 1 and 32% had an mUPD genotype. Two Italian patients were aged under 18 years (14.3 and 17.7 years), no other pediatric cases were reported. Obesity was present in 47%, type 2 diabetes mellitus in 35% and hypertension in 29% of patients, all being adults. One-third of patients took psychotropic medication that is known to cause SIADH (carbamazepine, SSRI antidepressant), one-quarter had EFI and, in 12% of cases, the hyponatremia was likely due to hyperglycemia. No cause of hyponatremia was found in 29%, including the two children (neither thyroid nor cortisol insufficiency), but SIADH was not excluded (no urine electrolyte or osmolality measurements in 30 out of 34 patients).

There was no specific management in 38% of these patients. Fluid restriction was introduced in 24%, medication (psychotropics or antihypertensive treatments) was changed in 18% and improvement of glycemic control was sought in 18% of patients (Table 2).

## 4. Discussion

We reported the largest cohort of patients with PWS with a history of hyponatremia to identify the possible causes of hyponatremia and interventions to prevent the development of severe hyponatremia. In our study with data from seven countries, severe hyponatremia was rare (0.5%), but moderate or mild cases were more frequent, occurring in 2.6% of patients with PWS. While this prevalence is low, it is relevant in this vulnerable population. The variability of the prevalence of non-severe hyponatremia among countries can probably be explained by the variability of the frequency of routine serum sodium assessment in patients with PWS among countries.

In our cohort, the prevalence of severe hyponatremia was higher in patients with mUPD (5 out of 7 patients), probably due to an increase in EFI and the presence of psychotropic medication that may promote SIADH. Indeed, behavioral problems and psychiatric diagnoses, such as psychosis, are more common in patients with the mUPD genotype [16]. In agreement with our findings, a previous study reported severe hyponatremia in two adults with PWS due to mUPD, in whom the precipitating factors were EFI and treatment with psychotropic medication known to induce SIADH [12]. In another report of severe hyponatremia in an adult with PWS, water intoxication due to desmopressin treatment for nocturnal enuresis was implicated [13]. In our study, apart from the direct effect of desmopressin treatment in two adults, the etiology of the severe hyponatremia in the other adults with PWS was less clear and likely based on several factors, including EFI and treatment with psychotropic medications known to be associated with SIADH.

Similarly, the etiology of mild–moderate hyponatremia in our cohort was often multi-factorial. Type 2 diabetes mellitus was almost twice as common in this cohort (35% of patients) than the rate of 20% reported in European adults with PWS [17,18,19]. In 12% of those with mild–moderate hyponatremia, hyperglycemia was present and these patients may be more prone to glucose-induced hyponatremia than the general population. While we did not find the cause of non-severe hyponatremia in 29% of patients, whether they displayed SIADH is not known as urine electrolytes and osmolarity were not measured.

These unexplained cases of hyponatremia could suggest a heightened sensitivity of the hypothalamic nuclei to the over-secretion of ADH in PWS. Given the numerous central endocrine abnormalities in this syndrome [9], a dysregulation of hypothalamic nuclei, both spontaneously and by drugs, is probable and could be contributing to the increased prevalence of hyponatremia in PWS. In the postmortem study of Swaab et al., the number of vasopressin neurons in the hypothalamic paraventricular nucleus of the five cases with PWS was not significantly different from 27 controls, whereas the number of oxytocin neurons was lower in the PWS cases compared to the controls [20]. Although the number of vasopressin cells is not apparently increased in PWS, the density of vasopressin immunostaining was not examined and there may still be over-secretion of vasopressin. Alternatively, increased renal sensitivity to vasopressin in PWS could be another explanation, though this is difficult to confirm in clinical practice.

Finally, EFI, alone or combined with other causes, was the most frequent etiology of hyponatremia in our cohort, where it was present in up to 15% of adults with PWS in a previous Swedish study [12]. While the majority of infants with PWS drink an unusually small amount, as they age, episodes of consumption of excessive amounts of water can occur and water intoxication was a frequent cause for hospitalization in Dutch adults with PWS in one study [21]. EFI is likely to be exacerbated by psychiatric medications, many of which result in mouth dryness, which is compounded by the sticky saliva of people with PWS [22]. It is thus important that individuals with PWS, their parents and their caregivers are well informed about this health hazard; the PWS Association USA has published a “water intoxication alert” on their website [23]. Fluid intake in individuals with PWS should always be monitored because of the risk of it becoming excessive with the subsequent development of hyponatremia.

The other unambiguous cause of severe or non-severe hyponatremia is desmopressin treatment in adults with nocturnal enuresis. If this treatment is instituted, fluid intake should be restricted for at least one hour before and eight hours after taking the treatment [24]. However, as water intake is difficult to control in most adults with PWS, desmopressin treatment should be avoided in adults with PWS. In cases of enuresis, patients should be comprehensively reviewed to find the exact mechanism(s) of enuresis and an alternative treatment to improve it [25].

Our study has limitations. The first is the retrospective design of the study. The second is the heterogeneity of the cases between countries. The last one is the absence of the comparison of data due to the absence of accurate retrospective data on the group of patients with PWS without a history of hyponatremia.

## 5. Conclusions

Severe hyponatremia is rare in PWS (0.5%) but is potentially life threatening, with two deaths in our cohort. Even if there are often multiple causes, the prevalence of severe hyponatremia increases in individuals with mUPD, in whom EFI appears to be more common, and is compounded by the use of psychotropic treatments that can induce SIADH, as well as the use of desmopressin treatment. In our opinion, desmopressin should not be used to treat nocturnal enuresis in adults with PWS because EFI is frequent in these patients and not easily prevented. In addition, treatments that are known to promote SIADH should be used carefully in patients with PWS, especially carbamazepine and SSRI antidepressants. Finally, we recommend that serum sodium should be measured regularly in individuals with PWS, especially in those who demonstrate EFI or take psychotropic drugs that may promote SIADH.

## Figures and Tables

**Table 1:** Description of severe cases of hyponatremia in adults with Prader–Willi syndrome from three countries, possible causes and evolution.

	Country	Gender, Age (years), Genetic Diagnosis	Clinical Symptoms	Serum Sodium (mmol/L)	Urinary Osmolarity (mOsmol/kg)	Urinary Sodium (mmol/L)	Possible Causes	Evolution
Case 1	France	Male, 41, mUPD	Coma (seizures)	115	-	-	Desmopressin for nocturnal enuresis (Minirin Melt ^®^ 240 µg/d for 3 days)	Recovery; no recidivism after stopping desmopressin intake
Case 2	France	Male, 27, mUPD	Coma (seizures)	119	169	25	Excessive fluid intake and diarrhea for 3 days	Recovery; no recidivism with fluid restriction (1.5 L/d)
Case 3	France	Female, 23, mUPD	Coma (seizures)	115	482	75	Hidden intake of desmopressin (treatment stopped for several months)	Recovery; no recidivism
Case 4	France	Male, 31, mUPD	Coma	112	-	-	Possible excessive fluid intake in few hours during hospitalization in orthopedics	Death (cerebral edema due to severe hyponatremia)
Case 5	The Netherlands	Male, 55, mUPD	Seizures	121	-	-	Unknown	Death (probably due to inhalation of gastric content)
Case 6	The Netherlands	Female, 29, Del	Seizures	Unknown *	-	-	Treatment with furosemide and salt restriction for cardiac failure	Recovery (stopped furosemide)
Case 7	United Kingdom	Female, 39, Del	Confusion	122	149	37	Central adrenal insufficiency and possible SIADH due to sertraline (but no regression after stopping sertraline)	Recovery; fluid restriction necessary (2 L/d) to maintain normal serum sodium

mUPD: maternal uniparental disomy; Del: deletion; SIADH: syndrome of inappropriate antidiuretic hormone. * Serum sodium was not available for this case but hyponatremia was mentioned in the medical report.

**Table 2:** Cases of mild or moderate hyponatremia in patients with Prader–Willi syndrome from seven countries.

Characteristics of patients with mild or moderate hyponatremia (*n* = 34)	
Countries	France: 1; Spain: 1; Australia: 3; United Kingdom: 3; Sweden: 4; The Netherlands: 10; Italy: 12
Serum sodium (mmol/L)	131.4 ± 2.7 (124–134)
Absence of symptoms of hyponatremia (%)	33 (97)
Age (years)	35.8 ± 10.2 (14.3–55)
Gender (%)	Female: 17 (50); male: 17 (50)
Genetic subtype (%)	Deletion: 18 (53); mUPD: 11 (32); ICD: 1 (3); unknown: 4
Body mass index (kg/m^2^)	32.2 ± 8.7
Obesity * (%)	16 (47)
Type 2 diabetes (%)	12 (35)
Type 1 diabetes (%)	1 (3)
Hypertension (%)	10 (29)
Possible causes of hyponatremia (%)	
Excess fluid intake (EFI)	8 (24)
Desmopressin treatment	2 (6)
Psychotropic treatment	11 (32)
*-Carbamazepine*	*6 (18)*
*-SSRI antidepressant (Fluoxetine, Citalopram)*	*5 (15)*
Diuretics	3 (9)
*-Bumetanide*	*1 (3)*
*-Hydrochlorothiazide*	*2 (6)*
Hyperglycemia	4 (12)
Unknown cause	10 (29)
Management (%)	
Fluid restriction	8 (24)
Reduce or stop treatment causing SIADH	4 (12)
Change of antihypertensive treatment	2 (6)
Improve glycemic control	6 (18)
No specific management	13 (38)

Results are expressed as mean ± SD (range) for continuous variables and as number (percentage) for categorical variables. mUPD: maternal uniparental disomy. ICD: imprinting center defect. BMI: body mass index. SSRI: selective serotonin reuptake inhibitor. SIADH: syndrome of inappropriate antidiuretic hormone. * Obesity was defined as BMI ≥ 30 kg/m^2^ in adults and a BMI Z-score ≥ 3 in children.

## Data Availability

The data are not publicly available due to privacy and ethical restrictions. The data that support the findings of this study are available on request from the corresponding author.
